# Prosocial Behavior Increases with Age across Five Economic Games

**DOI:** 10.1371/journal.pone.0158671

**Published:** 2016-07-14

**Authors:** Yoshie Matsumoto, Toshio Yamagishi, Yang Li, Toko Kiyonari

**Affiliations:** 1 Brain Science Institute, Tamagawa University 6-1-1 Tamagawagakuen, Machida, Japan; 2 Graduate School of International Corporate Strategy, Hitotsubashi University, Tokyo, Japan; 3 School of Social Informatics, Aoyama Gakuin University, Sagamihara, Japan; Technion Israel Institute of Technology, ISRAEL

## Abstract

Ontogenic studies of human prosociality generally agree on that human prosociality increases from early childhood through early adulthood; however, it has not been established if prosociality increases beyond early adulthood. We examined a sample of 408 non-student residents from Tokyo, Japan, who were evenly distributed across age (20–59) and sex. Participants played five economic games each separated by a few months. We demonstrated that prosocial behavior increased with age beyond early adulthood and this effect was shown across all five economic games. A similar, but weaker, age-related trend was found in one of three social value orientation measures of prosocial preferences. We measured participants’ belief that manipulating others is a wise strategy for social success, and found that this belief declined with age. Participants’ satisfaction with the unilateral exploitation outcome of the prisoner’s dilemma games also declined with age. These two factors—satisfaction with the DC outcome in the prisoner’s dilemma games and belief in manipulation—mediated the age effect on both attitudinal and behavioral prosociality. Participants’ age-related socio-demographic traits such as marriage, having children, and owning a house weakly mediated the age effect on prosociality through their relationships with satisfaction with the DC outcome and belief in manipulation.

## Introduction

Do individuals’ general inclinations for prosociality and cooperativeness increase with age throughout life? This seemingly simple question has escaped systematic investigation, and the few studies that have investigated this issue have produced inconsistent findings. Most studies concerning age-related changes in prosociality have focused on childhood, that is, the change from early childhood to early adulthood [1–12]. These studies have typically shown that prosociality increases during childhood [1–8]; however, some exceptions have been reported [9, 10]. Although rejection of unfair offers in an ultimatum game often decrease with age [11, 12], rejection of unfair offers in an ultimatum game may not qualify as prosocial behavior [13, 14]. However, whether prosociality increases with age beyond early adulthood has not been established [9, 12, 15]. Van Lange and colleagues [15] conducted a study that measured participants’ prosocial attitude (i.e., social value orientation; SVO) using a large national sample from the Netherlands (*N* = 1,728), including responders whose age ranged from 15 to 89 years. SVO corresponds to relatively stable preferences for the distribution of resources for oneself and others [16, 17], and a meta-analysis showed that it is correlated with actual cooperative behavior in the prisoner’s dilemma game (PDG) (approximately *r* = .3) [18]. Van Lange and colleagues [15] found that the triple-dominance measure (TDM) of SVO prosociality increased from early adulthood to middle and old age. These researchers suggested two hypotheses, not necessarily mutually exclusive, for the positive effect that age has in promoting prosociality [15]. The first is the individual learning hypothesis that individuals learn the positive consequences of acting in prosocial manners either directly or vicariously as they accumulate life experiences. Thus, individuals behave prosocially when they detect cues suggesting interdependence with others (including economic game situations). The second is the situational change hypothesis that the nature of social interactions people face changes as the social roles they play in their lives change with age. In addition to the study of SVO, a study by Van den Assem and colleagues [19] showed an increase in prosocial behavior among men using data of the contestants’ choices in a British TV program called “Golden Balls.” The game was a variant of the PDG where defection weakly dominated cooperation. On the other hand, a study by Gutiérrez-Roig and colleagues [20] found no age difference in cooperation rate in a public goods game, except for young children, who displayed a significantly lower level of cooperation than the rest, and older people over 65 years, who displayed a higher level of cooperation than the rest. Due to the relatively small size (*N* = 168) and the non-standard nature of the sample consisting of volunteers who were recruited at a board game festival, a direct comparison of this study with earlier studies is difficult. Another difficulty in comparing the studies that reported a positive effect of age [15] and those that reported no effect [19] concerns the measures of participants’ prosociality. Van Lange’s study used a well-established measure of SVO, which correlated with actual cooperative behavior [15]. Gutiérrez-Roig’s study used the actual cooperation choices in an iterated 4-person public goods game. It is possible that age is differently related to these two types of measures: attitudinal measures of prosocial preferences (SVO prosociality) and actual cooperative choices in an economic game. We further noticed that the earlier studies mentioned above were all conducted with Western European samples including the Netherlands national sample; therefore, it is not clear how these findings and conclusions are generalizable beyond the Western culture.

Facing the paucity of reliable data showing the relationship or lack thereof between age and prosociality and possible Western bias in earlier studies, we addressed whether behavioral and attitudinal prosociality increases with age using data obtained from a large-scale research project with 564 initial participants (age range = 20–59) from Japan. This research project was launched in 2012 and it has been conducted in 8 waves since the end of 2015. We used the overall measure of prosocial behavior based on five economic games participants played, most of which were conducted in different waves to minimize carry-over effects. We also measured participants’ SVO in three waves, each time with a different method to ensure generalizability of findings beyond a particular method. In addition to these two sets of major variables and age, we used the following individual difference measures that would help us understand the age-prosociality relationship. The first set of measures consisted of those that would help us understand the aspects of prosociality that are related with age. The SVO measure of prosociality has been known to represent a combination of preferences for the joint gain and equality [21]; therefore, it is useful to know what aspect of prosociality is more strongly related with age. The Slider Measure (SLM) [22] of SVO prosociality provides subscales that separately measure preferences for joint gain and equality. The other two measures, the TDM [15] and the Ring Measure (RGM) [23], cannot be used to separate the two. In addition to the subscales of SLM, we used participants’ satisfaction with the four possible outcomes of the PDG, which they reported in the post-experimental questionnaire after the first and the second PDGs. The second set of measures was the scales that were constructed to measure participants’ beliefs about life strategies that were instrumental for social success. We used these measure to assess if the age-related changes in prosociality would be solely related to change in preference or also involve additional changes in beliefs that prosocial or proself behavior would be instrumental for social success. The individual learning hypothesis proposed by Van Lange and colleagues [15] predicts that age is more directly related to changes in such beliefs than to changes in preferences. The third set of measures was the participants’ demographic traits. We included these measures in our analysis to assess if the age-prosociality relationship we might find would be specific to particular types of individuals. Based on the analysis of these variables, we found a significant and substantial correlation between age and prosocial behavior and a weaker, but significant, correlation with one of the three measures of SVO prosociality. The correlation of age and prosocial behavior was not considerably affected after controlling for the three SVO measures of prosociality. The positive effects of age on both attitudinal and behavioral prosociality were mediated by satisfaction with the unilateral defection outcome of the PDGs and the belief that manipulating others was a wise strategy for success.

## Methods

The study protocol was approved by the Ethics Committee of the Brain Science Institute at Tamagawa University, where the study was conducted according to the approved protocol, and met the requirements of the Declaration of Helsinki. An informed consent form was signed by each participant.

### Sample

This study analyzed data obtained in a large research project, which continued over a period of four years. Initially, 600 individuals from a suburban area of Tokyo were selected from approximately 1,700 applicants who responded to invitation brochures distributed to approximately 180,000 residents. The selection of participants was determined to include the same number of participants by age and sex (75 men and 75 women in each 10-year age group). Of the 600, 564 actually participated in the initial wave of this study (May–July 2012) and repeatedly participated in the following seven waves with some temporary or permanent dropouts. (See Figs A-H in S2 File for distributions of the participants’ socio-demographic traits.) The study was conducted in eight waves between 2012 and 2015, each separated by a few months. Among the 564 participants, we analyzed data from 408 participants who participated in all five economic games. These 408 participants’ distribution across major demographic variables is shown in Figs A-H in S2 File. The dataset that was generated by this large research project has been used in publications on the topics of *Homo economicus* [24], construction of trust scales [25], the relationship between oxytocin and trust [26], and strategic behavior and brain structure [27]. None of the previous publications based on this dataset focused their analysis on the relationship between age, behavioral and SVO prosociality.

### The economic games behaviors

We used game behaviors in five economic games: a repeated one-shot prisoner’s dilemma game (wave 2), a one-shot prisoner’s dilemma game (wave 4), an n-person social dilemma game (waves 4), a dictator game (wave 3), and a trust game (return choice) (wave 5) to construct the overall behavioral measure of prosociality). See S1 File for further information about these 5 games.

#### Prisoner’s dilemma game I: repeated one-shot game

Participants decided whether they would provide an endowment to their partner or keep it for themselves. When the endowment was provided, the partner received twice the amount of the endowment. Each participant played the game for nine trials, each time with a unique combination of the endowed size (JPY 300, 800, or 1,500), and the protocol (simultaneous protocol, first player in the sequential protocol, and second player protocol). The participants were instructed and actually paid for three of the nine trials. The randomly matched partner made the same decision. We used the proportion of trials that the participant provided his or her endowment to the randomly matched partner as an indicator of prosocial behavior in the prisoner’s dilemma game I, excluding the participant’s responses to the first player’s defection in the second player trials because only a few of the participants cooperated in these trials.

#### Prisoner’s dilemma game II: one-shot game

The one-shot PDG with the simultaneous protocol was used. The participants were endowed with JPY 1,000 and they decided how much of it they would provide to their partner in increments of JPY 100. When some of the endowment was provided, the partner received twice the amount. The portion of the endowment the participant did not provide was the participant’s to keep. The randomly matched partner made the same decision. We used the proportion of endowment the participant provided to his or her partner as an indicator of prosocial behavior in prisoner’s dilemma game II.

#### Dictator game

All participants first played a one-shot dictator game as dictators with a randomly matched recipient, expecting that half of them would be assigned to the role of recipients. Each participant was given an endowment of JPY 1,000 and decided how much of the endowment to provide to their partner (the recipient). Following the initial dictator game, participants played similar games six times as a dictator, with a different recipient each time. The size of the endowment varied each time, ranging from JPY 300–1,300 (i.e., 300, 400, 600, 700, 1,200, and 1,300). Participants were told that they would play the game an unspecified number of times. All participants made allocation decisions as a dictator in each game first, and then were randomly assigned either the role of dictator or the recipient. We used twice the mean proportion of endowment that the participant allocated to his or her partners as an indicator of prosocial behavior in the dictator game because providing 50% of the endowment was the fair choice for the dictator. When the mean proportion exceeded .5, we set the participant’s prosociality indicator in the dictator game at 1, the same level of fair choice as those who provide 50% of the endowment. The additional analysis with the original score rather than the truncated score did not affect the conclusions.

#### Social dilemma game I and II

The same design was used in the two social dilemma experiments. The instruction was written for a 10-person group; however, the participants were told that the actual group size could vary. The game was played once. Each participant was given an endowment of JPY 1,000 and decided how much of it to provide for the production of a public good in increments of JPY 100. The sum of the provided money was doubled and equally allocated to all members regardless of their provision level. We used the proportion of the endowment that the participant provided as an indicator of prosocial behavior in the social dilemma game.

#### Trust game

The trust game was played between two randomly matched participants: a truster and a trustee. The truster was provided with JPY 1,000 by the experimenter and decided how much of it to transfer to the trustee in increments of JPY 100. The transferred money was then tripled and provided to the trustee. The trustee received three times the transferred money and then decided how much of it to transfer back to the truster. All participants first played as trusters and decided how much of the JPY 1,000 to transfer to the trustee, and then played as trustees and made decisions using the strategy method. Finally, pairs of participants were formed randomly, one person from each pair was randomly assigned as either a truster or a trustee, and they received their payment according to the pair’s decision. We used the mean return proportion of the tripled money the participant transferred back (truncated at 50% as in the dictator game) as an indicator of prosocial behavior in the trust game.

### The overall measure of prosocial behavior

We decided not to include the second social dilemma game in the overall measure of prosocial behavior because its inclusion would have reduced the number of participants to be used in the analysis from 408 to 358 due to the large number of participant dropouts. The 5-game measure and the 6-game measure were highly correlated with each other at *r* = .99 (*p* < .0001). Participants’ choices were first standardized within each game (including participants who played the game but did not play some of the other games), and the overall measure of prosocial behavior was constructed by taking the mean of the standardized scores of the 5-game behaviors (Cronbach’s α = .85). To facilitate interpretations of the finding, we standardized the overall measure of prosocial behavior with a mean of 0 and standard deviation of 1 with the 408 participants who played all five games. The distribution of this overall prosocial behavior is shown in Fig I in S2 File.

### The SVO measure of prosociality

Participants’ SVO prosociality was measured three times, each time using a different method: the TDM [15] (wave 3), the RGM [23] (wave 6), and the SLM [22] (wave 5). Each measure of SVO prosociality consisted of a set of alternative ways to unilaterally allocate an imaginary reward between the participant and another individual (see S1 File for the specifics of the three measures). Participants were categorized in the RGM and the TDM either as prosocial or proself according to the respective methods used in previous studies [15, 23]. They were assigned a value between -16.3 (least prosocial) and 61.4 (most prosocial) according to the SLM [22]. The SLM also provided the responder’s preferences for the joint gain and equality for those who show preferences for prosociality.

### Satisfaction with the four outcomes of the PDG

In addition to the measures of SVO, we examined what aspects of the participants’ prosociality were responsible for the age effect by measuring participants’ satisfaction with each of the four cells in the PDG conducted in waves 2 (PDG-I) and 4 (PDG-II): the CC outcome where both partners cooperated, the DC outcome where the participant exploited a cooperative partner, the CD outcome where the cooperative participant was exploited by a non-cooperative partner, and the DD outcome where both players did not cooperate. Participants’ responses were measured in each game using a 7-point Likert scale ranging from 1 (*felt extremely unpleasant*) to 7 (*extremely happy*). We used the mean response of the two games in our analysis.

### Beliefs in strategies for social success

To measure participants’ beliefs about the strategies on how to succeed in life, we constructed the “strategy for social success scale” consisting of five subscales: manipulation, nepotism, honesty, risk avoidance, and assertiveness. Each of these subscales is presented in Table E in S1 File. The manipulation scale consists of six items (Cronbach’s α = 0.80) representing the belief that cheating, manipulating, and taking advantage of others is critical for achieving success in life. The nepotism scale consists of five items representing the belief about the importance for creating and maintaining strong relations with and being liked by people who would help them a (α = 0.82). The honesty scale consists of five items representing the belief that honesty is the best strategy for social success (α = 0.75). The risk avoidance scale consists of 5 items representing the belief that avoiding risks is the key to social success (α = 0.66). The assertiveness scale consists of five items representing the belief that having a firm conviction and asserting one’s self is the key to social success (α = 0.83).

### Social and demographic traits

To examine if the age-related changes in prosociality may be mediated or modulated by the age-related changes in social and demographic traits, we assessed each participant’s sex (48% female), subjective social class, annual income, college education, marital status, house ownership, number of children, and number of siblings. See Figs A-H in S2 File for distributions of these variables.

### Statistical analysis

The relationships between age and overall prosocial behavior and SVO prosociality were analyzed with Pearson correlations. When the analysis involved a binary dependent variable, we reported the point-biserial correlation for the descriptive purpose and Wald *χ*^2^ value for significance testing. For multi-variable analyses of behavioral or attitudinal prosociality, we used an ordinary least square regression analysis. We use the Sobel test for the mediation analysis.

## Results

### Age effect on prosociality

**Fig 1:**
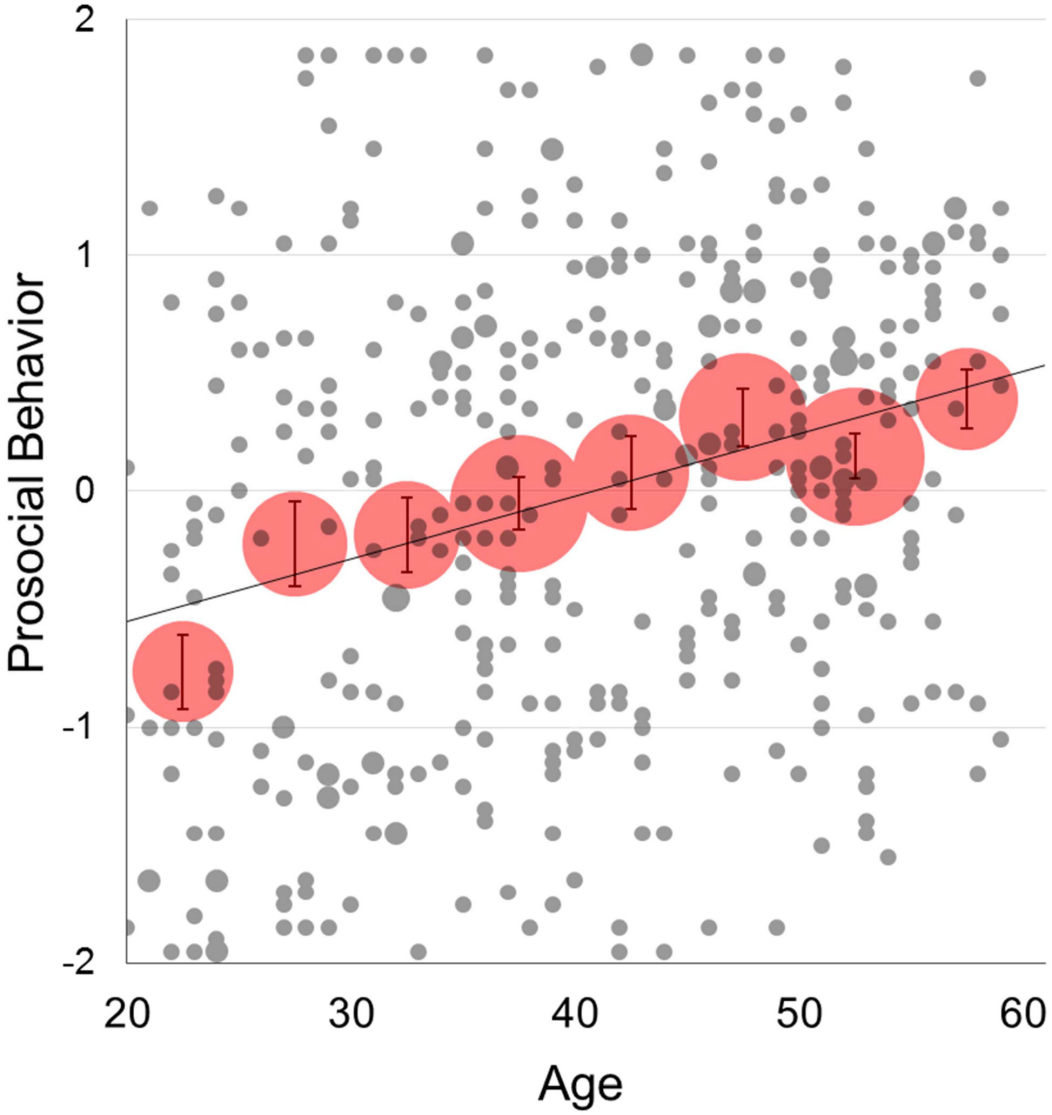
Relationships of age with overall prosocial behavior. Each gray circle corresponds to an individual participant’s prosocial behavior, and each red circle represents the 5-year mean. The size of each gray circle indicates the number of the same age participants who had the same prosocial behavior score, and each red circle indicates the sample size for each 5-year age range. Error bars represent standard errors.

**Fig 2:**
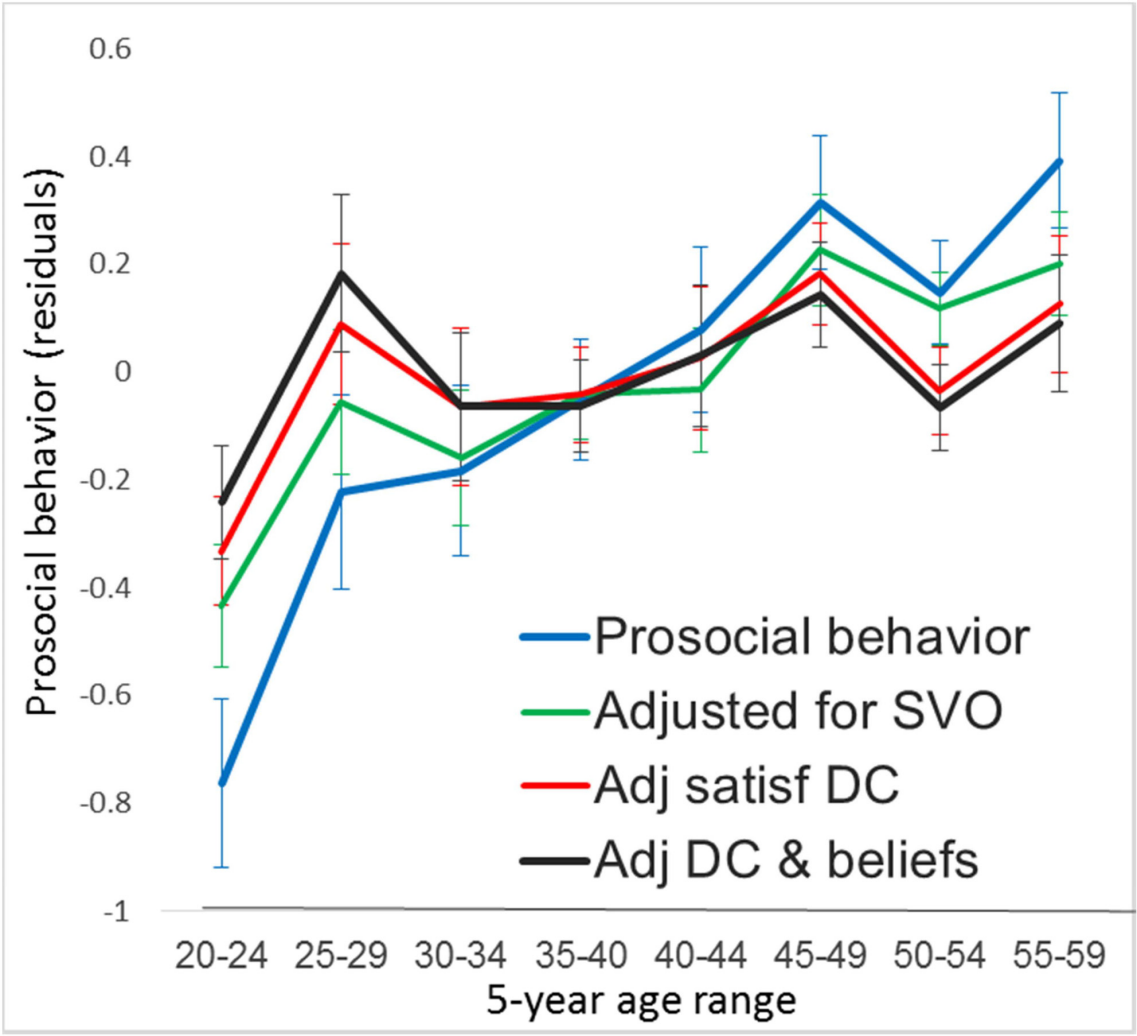
Relationships between age and prosocial behavior. The positive relationship between age and prosocial behavior (blue line) is maintained after controlling for SVO prosociality (adjusted for SVO, green line) or satisfaction with the DC outcome (adjusted satisfaction, red line). The relationship ceases to be significant when the satisfaction of the DC outcome and the belief in manipulation are controlled (adjusted satisfaction and belief, black line).

We used participants who participated in all five economic games in the following analysis (N = 408). Fig 1 indicates a positive relationship between age and prosocial behavior (*r* = .28, *p* < .0001). A similar positive relationship was found with each of the five constituent games: *r* = .19, *p* < .0001 (PDG-I); *r* = .20, *p* < .0001 (PDG-II); *r* = .28, *p* < .0001 (DG); *r* = .15, *p* = .002 (SDG); and *r* = .28, *p* < .0001 (TG). The average levels of prosocial behavior across age groups are also depicted in Fig 2 (blue line). Although the blue line in Fig 2 suggests a non-linearity of this relationship, the quadratic effect in a regression analysis did not reach significance level (*β* = -0.00075, *SE* = 0.00046, *t* = 1.63, *p* = .104). Despite the fact that the three measures of SVO prosociality were correlated with each other (*r*_TDM.SLM_ = .47, *p* < .0001; *r*_TDM.RGM_ = .33, *P* < .0001; *r*_SLM.RGM_ = .42, *p* < .0001) and that each was correlated with prosocial behavior (BEH)(*r*_TDM.BEH_ = .43, *p* < .0001; *r*_SLM.BEH_ = .66, *p* < .0001; *r*_RGM.BEH_ = .39, *p* < .0001), only the SLM was significantly correlated with age (*r*_TDM.AGE_ = -.02, *p* = .630; *r*_SLM.AGE_ = .17, *p* < .001; *r*_RGM.AGE_ = .04, *p* = .439). These findings only partially replicate the earlier finding of a positive relationship between age and SVO prosociality [15]. Given this unexpected inconsistency in the relationship between age and the three measures of SVO prosociality, we decided to focus our analysis of SVO prosociality on the SLM by dropping the other two measures from further analysis. While prosocial behavior was strongly related with the SLM prosociality, the relationship between age and prosocial behavior remained significant when SLM prosociality was controlled (*r*_p_ = .23, *p* < .0001). The green line in Fig 2 shows a steady increase in the residual prosocial behavior even after controlling for SLM prosociality.

**Fig 3:**
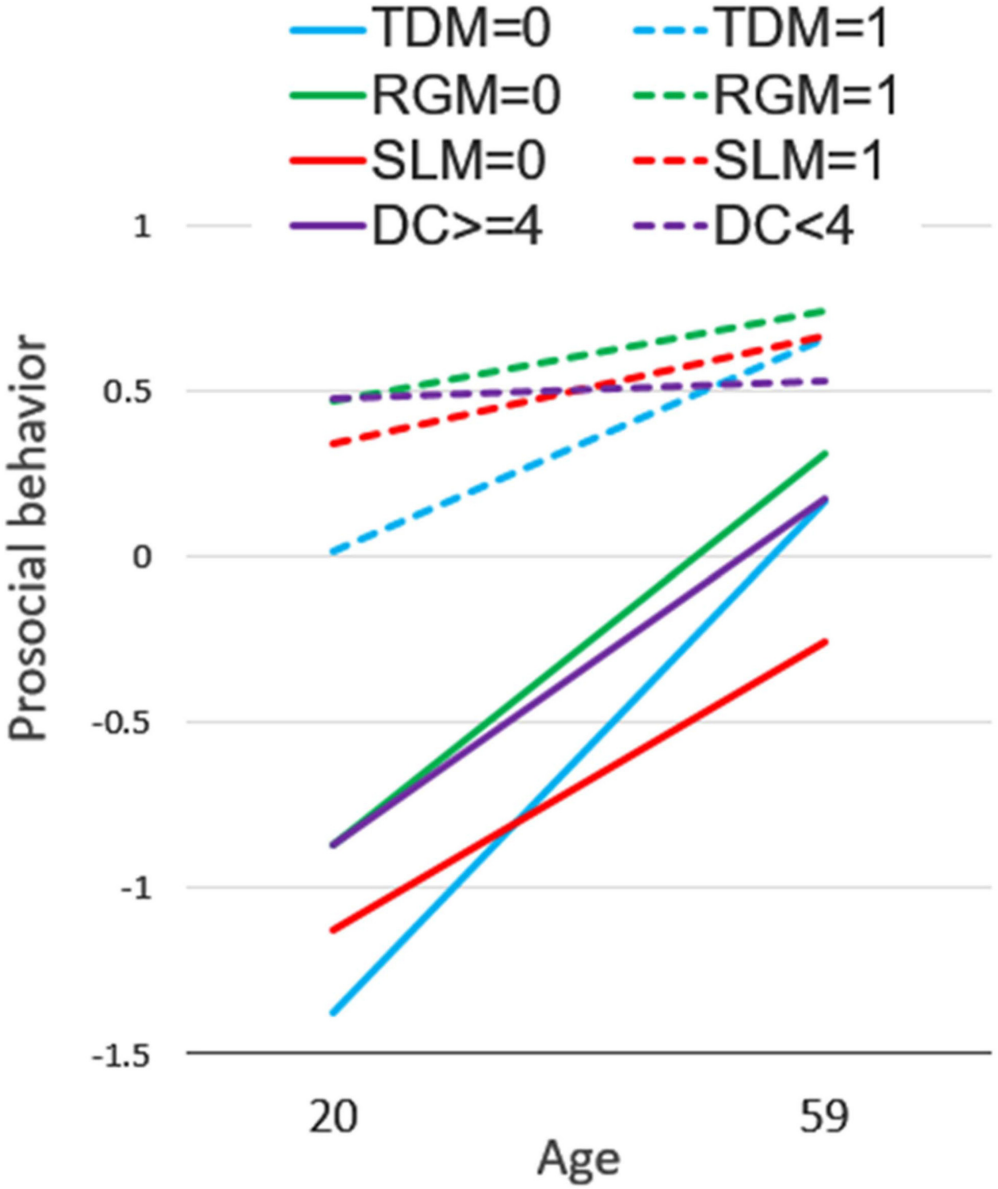
Regression lines each representing the effect of age on prosocial behavior for a level of the three SVO measures, and satisfaction with the DC outcome. These lines represent regression lines obtained from the regression equations including both the main and the interaction effects. The SLM was dichotomized to proselfs and prosocials in this figure, and so are satisfaction (below or above the scale mid-point of 4).

We further explored if age’s effect on prosocial behavior would interact with SVO prosociality. Age interacted with the TDM (*F*(1,380) = 7.23, *p* = .008) and the RGM (*F*(1,362) = 5.43, *p* = .020). The interaction was not observed with the SL measure of SVO (*F*(1,404) = 0.83, *p* = .364), but was marginally significant when the participants were categorized to proselfs and prosocials [22] (*F*(1,404) = 3.60, *p* = .059). As shown in Fig 3, the age effect was stronger among proselfs than prosocials, suggesting that the increase in prosocial behavior takes place mostly among proselfs. That is, even proselfs behave more prosocially as they age.

### Satisfaction with the four outcomes in the PDG

**Fig 4:**
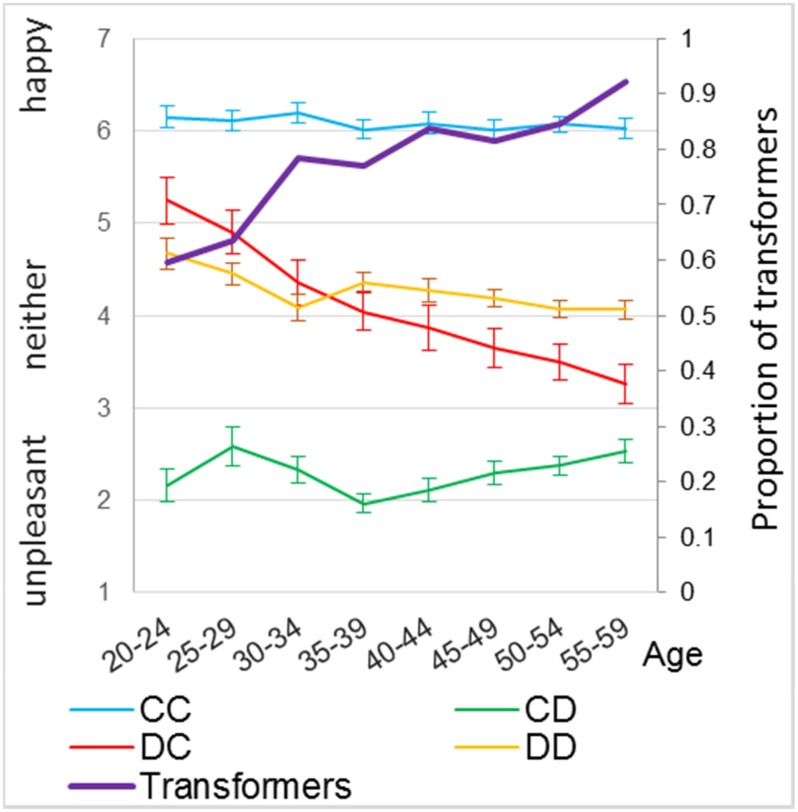
The relationship between satisfaction with the four PDG cells and age (in 10-year intervals). Fig 4 shows the levels of happiness vs. unpleasant for the CC outcome (blue line), for the DC outcome (red line), for the CD outcome (green line), and for the DD outcome (orange line). Transformers refer to the proportion of the participants who have subjectively transformed the PDG to a coordination game. Error bars represent standard errors.

The SLM provided separate measures for joint gain and equality for those who were classified as prosocials; however, either the preference of joint gain (*r* = -.00, *p* = .976) or of equality (*r* = -.04, *p* = .561) was not correlated with age among the participants who were categorically classified as prosocials. This lack of correlation with joint gain or equality seems to reflect the fact that the effect of age on prosociality involved the contrast between prosocials and proselfs rather than the subtle difference between preferences for joint gain or equality among prosocials. Concerning satisfaction with the four outcomes in the PDGs, which all participants including prosocials and proselfs responded, satisfaction with the unilateral defection (DC) outcome was most strongly correlated with prosocial behavior (*r = -*.60, *P* < .0001), followed by satisfaction with the mutual defection (DD) outcome (*r = -*.31, *p* < .0001), and the mutual cooperation (CC) outcome (*r* = .29, *p* < .0001). Satisfaction with the victim outcome (CD) where the player cooperated and the partner defected was not correlated with prosocial behavior (*r* = .08, *p* = .114) because almost everyone including both behaviorally prosocials and proselfs disliked being exploited by uncooperative partners (Fig 4). Among the four outcomes, only the participants’ satisfaction with DC and DD cells significantly correlated with age (*r* = -.34, *p* < .0001, and *r* = -.18, *p* < .001, respectively) (Fig 4 and Table 1). The participants’ preferences for the other two cells, CC and CD, were not significantly related with age (Table 1). When satisfaction with the DC outcome and the DD outcome were simultaneously entered as independent variables together with age in a regression analysis of SLM, satisfaction with the DC outcome had a significant effect (*β* = -4.099, *t* = 9.73, *p* < .0001), while satisfaction with the DD outcome did not (*β* = -1.044, *t* = 1.30, *p* = .195). The effect of age ceased to be significant (*β* = 0.005, *t* = 0.08, *p* = .938). Satisfaction with the DC outcome alone almost completely mediated the age effect on SLM (Sobel test, *t* = 6.04, *p* < .0001); when satisfaction with the DC outcome alone was controlled, the effect of age on SLM prosociality became non-significant (*β* = 0.014, *t* = 0.21, *p* = .835). Satisfaction with the DC outcome also mediated the effect of age on prosocial behavior. When it was controlled, the correlation between age and prosocial behavior was reduced from *r* = .28 to *r*_p_ = .10 (*p* = .037). The red line in Fig 2 represents the residual effect of age on prosocial behavior after controlling for satisfaction with the DC outcome. The mediation effect of satisfaction with the DC outcome was significant (Sobel test, *t* = 6.51, *p* < .0001).

**Table 1:** Correlations between age, SVO prosociality, prosocial behavior, and satisfaction with the four cells in the prisoner’s dilemma games.

Outcome (player’s choice, partner’s choice)	Mean (SE)	With—SLM ^a^	With prosoc behavior ^b^	With Age
CC	6.072 (0.038)	.261****	.288****	-.056
DC	4.023 (0.083)	-.479****	-.595****	-.338****
CD	2.272 (0.049)	.100*	.078	.051
DD	4.256 (0.042)	-.184***	-.305****	-.181***

*p < .05

***p < .001

****p < .0001

^a^SVO prosociality

^b^prosocial behavior

Satisfaction with the DC outcome also interacted with age (*F*(1,404) = 6.48, *p* = .011) in such a way that age had a stronger effect on prosocial behavior among those who were satisfied with the DC outcome than those who felt unpleasant with the same outcome (Fig 3). Again, it is suggested that those who feel happy with earning as much as they could at an expense of the interaction partner are the ones who become to behave prosocially as they age.

One way to interpret satisfaction with the four outcomes is through its relation with the way participants subjectively construed the game. The majority (78.4%) of participants stated that they were more satisfied with the CC outcome than the DC outcome despite the fact that their monetary rewards were higher in the latter than the former. In the subjective evaluation of the satisfaction of outcomes, including their own benefits and those of the partner, the majority of participants played the PDG as if it were an assurance game [28] or a stag-hunt game [29] when mutual cooperation yields a better outcome than unilateral defection. The proportion of these subjective “game transformers” [30] increased with age (*r* with age = .20, *p* < .0001; 61.5% in the 20s, 77.7% in the 30s, 82.6% in the 40s, and 87.2% in the 50s).

#### Beliefs in strategies for social success

Participants’ belief that manipulating others for their own benefit was a socially wise strategy negatively correlated with their prosocial behavior (*r* = -.33, *p* < .0001) and decreased with age (*r* = -.24, *p* < .0001). Similarly, the belief that establishing and maintaining nepotistic relations was a socially wise strategy negatively correlated with their prosocial behavior (*r* = -.22, *p* < .0001) and decreased with age (*r* = -.21, *p* < .0001). The belief that honesty was a good strategy for social success also correlated with prosocial behavior (*r* = .17, *p* < .001) and increased with age (*r* = .11, *p* = .032), but the correlations were weaker than those found in the previous two were. The belief that avoiding risks is a good strategy for social success was negatively correlated with prosocial behavior (*r* = -.18, *p* < .001), but it was not correlated with age (*r* = -.03, *p* = .526). The belief that being assertive was a wise strategy for social success was not significantly correlated with prosocial behavior (*r* = -.09, *p* = .077) or age (*r* = .01, *p* = .869). Controlling for the three beliefs that correlated both with prosocial behavior and age in addition to satisfaction with the DC outcome reduced the correlation between age and prosocial behavior to a non-significant level (*r*_p_ = .06, *p* = .216). The black line in Fig 2 represents the residual prosocial behavior after controlling for the satisfaction and beliefs. A regression analysis of prosocial behavior revealed that satisfaction with the DC cell (*β* = -0.303, *t* = 11.89, *p* < .0001) and belief in manipulation (*β* = -0.152, *t* = 3.19, *p* = .002) had significant effects. The belief in nepotism (*β* = -0.074, *t* = 1.52, *p* = .129), honesty (*β* = 0.106, *t* = 1.78, *p* = .077), or age (*β* = 0.005, *t* = 1.24, *p* = .216) did not. The belief in manipulation alone significantly mediated the age effect on prosocial behavior (Sobel test, *t* = 4.06, *p* < .0001).

### Socio-demographic variables

We finally examined whether the socio-demographic traits of the participants (see S1 File and Figs A-H in S2 File) mediated the effect of age on attitudinal and prosocial behavior. Most of the socio-demographic variables except sex and college education were significantly correlated with age. However, none of these variables mediated the effect of age on SVO prosociality or interacted with age. Marital status, number of children, and home ownership were significantly and positively correlated with both prosocial behavior (*r* = .14, *p* = .004; *r* = .12, *p* = .013; *r* = .10, *p* = .043, respectively) and age (*r* = .49, *p* < .0001; *r* = .52, *p* < .0001; *r* = .45, *p* < .0001, respectively), and significantly mediated the effect of age on prosocial behavior (Sobel test, *t* = 2.81, *p* = .005 for marital status; *t* = 2.46, *p* = .014 for number of children; *t* = 1.99, *p* = .047 for home ownership). When these three variables were controlled, the correlation of age and prosocial behavior was slightly reduced to *r*_p_ = .23, (*p* < .0001). However, when age, satisfaction with the DC outcome, belief in manipulation, marital status, number of children, and home ownership were simultaneously entered as independent variables in a regression analysis of prosocial behavior, none of the three demographic variables remained significant (*β* = 0.036, *t* = 0.34, *p* = .730 for marital status; *β* = -0.028, *t* = 0.61, *p* = .539 for number of children; and *β* = -0.127, *t* = 1.32, *p* = .188 for home ownership). The age-related changes such as getting married, having children and acquiring a house, indirectly made people more prosocial through decrease in the satisfaction with the DC outcome and the decrease in the belief that manipulating others is a successful life strategy. None of the socio-demographic traits had interaction effects with age on prosocial behavior. Correlations between all variables used in the study are reported in the S3 File.

## Discussion

We provided strong evidence that prosocial behavior increases with age even after people reach young adulthood. The first conclusion of this study is that people develop a prosocial behavioral pattern as they age, accompanied by a decline in the satisfaction in the outcome that they unilaterally exploit interaction partners and the belief that manipulating others is a wise strategy to be successful in life. These findings suggest that age-related changes in prosociality take place together with a change in focus from immediate to long-term gains. As suggested in an earlier study [15], people may learn through their life experiences that pursuit of immediate gains often leads to undesirable long-term consequences. A related finding worthy of discussion is that participants on average were more satisfied with the CC outcomes rather than the DC outcomes despite the fact that they could earn more money by unilaterally defecting on cooperative partners. This finding is consistent with earlier findings involving six PDG studies [31].

One interpretation of this finding is that the majority of participants played the PDG as if it were a stag-hunt (or assurance) game [28, 29], when it is more beneficial to cooperate than to defect insofar as the partner cooperates. In other words, our participants on average subjectively transformed [30–33] the PDG to a coordination game such as a stag-hunt (or assurance) game. While a substantial proportion of young participants in their 20s played the PDG as such (more satisfied with the DC outcome than the CC outcome), the overwhelming majority of the older participants played the PDG as a subjectively transformed stag-hunt game where CC can be an equilibrium.

This interpretation is consistent with the individual learning hypothesis proposed by Van Lange and colleagues [15] according to which the increase in prosociality with age is a reflection of direct or vicarious experiences of long-term negative outcomes of temporarily successful exploitation of interaction partners [15, 30]. People may have developed a heuristic to perceive social interactions involving a possibility of mutual cooperation as a coordination game rather than a prisoner’s dilemma game [31, 32]. This interpretation is also consistent with the shift in the situational change hypothesis also suggested by Van Lange and colleagues [15]. That is, older people may face real coordination games including repeated PDG in their social life more frequently than do younger people whose prospects of future life change provide true one-shot situations. While the possible avenues through which older people become more prosocial are likely intertwined and complex, it is nevertheless important to find ways to separate them in developing the explanation of the age effect on prosociality in future studies.

Satisfaction with the DC outcomes that was found to play a crucial role in mediating age with prosociality requires thorough investigation, both conceptually and empirically, in future studies. It can be interpreted in multiple ways. It may be considered a reflection of the player’s preference for (or aversion of) advantageous inequality in resource allocation because the DC outcome is maximally different between the player and the partner in favor of the player [34]. An alternative interpretation is that it is a reflection of the player’s moral commitment (or preference for being a morally righteous person). The third interpretation that is more consistent with the individual learning hypothesis is that older people learn from their life experiences to intuitively perceive the PDG as a stag-hunt game. The subjective transformation of the PDG into the assurance game has been characterized in earlier studies [31, 32] as the defining feature of the social exchange heuristic. The subjective game transformers are those who have acquired the social exchange heuristics that make them cooperate in social interactions insofar as the partner is expected to behave in a similar way. For them, unilateral defection is not an attractive option because they intuitively associate it with the long-term outcome of mutual defection. These possible interpretations are all speculations; however, future investigation of these possibilities will provide a firmer grip of the mechanisms through how people become more prosocial as they age.

We failed to exactly replicate an earlier finding [15] of a positive relationship between age and TDM prosociality. Problems with the Japanese version of the TDM cannot explain this result, because the Japanese TDM version was strongly correlated with prosocial behavior, confirming its high predictive validity with the Japanese sample, comparable with the Dutch sample. Although age was significantly correlated with one of the three measures of SVO, the SLM, this correlation was weaker than the age-behavior correlation. These findings suggest that behavior is more strongly affected by social exchange heuristics [31, 32, 35, 36] than preferences measured by SVO. This explanation is also consistent with the transformer interpretation of the DC outcome.

Furthermore, this could explain why age was strongly related with prosocial behavior even after controlling for the SLM, because this result indicates that the positive effect of age on behavior was predominantly a reflection of factors not related to preferences per se. Similar inferences were drawn from the findings that the age-related increase in prosocial behavior was evident among those sharing the same level of SVO prosociality, particularly among proselfs. People may not change their preferences over age, at least not as much in Japan as in the Netherlands. However, they may learn that behaving in a prosocial manner is the better strategy for their long-term adaptation.

Finally, it should be noted that our findings are based on the analysis of cross-sectional data, and deriving any causal inferences is problematic. Particularly problematic is the causal relationships between satisfaction with the DC outcome, reduction in the belief in manipulation, and prosocial behavior, which were all mutually correlated and changed with age. This study established an age-prosociality relationship that had not been clearly determined before and opened the door to a new stage of research to identify the mechanisms that produce this relationship. Another topic for future study concerns the generalizability of our findings beyond the particular socio-cultural backgrounds of our sample. We found that major demographic factors such as sex, marital status, subjective social class, income, college education, and home ownership did not strongly affect the relationship between age and prosociality. Furthermore, the previous finding by Van Lange and colleagues [15], based on a large national sample in the Netherlands, which showed that SVO prosociality increases with age, provides support to the conclusion that the current finding is likely not limited to a Japanese sample that is culturally distinct from Western populations. On the other hand, we failed to replicate an earlier finding [15] when the same TDM was used. Therefore, it is possible to speculate that age is related with changes in preferences more in the West and more with changes in heuristics in the East. The social-exchange heuristics that cause people to perceive even the one-shot PDG as if it is embedded in repeated interactions may be particularly strong in societies where repeated interactions with the same set of people are more prevalent, typically referred to as collectivist societies including the Japanese society, than societies where people can change interaction partners more easily. What needs to be examined in larger cultural and societal contexts is how generalizable the increase in subjective game transformers and reduction in the belief in manipulating others mediate the relationship between age and prosocial behavior.

## Supporting Information

S1 Dataset Dataset including all variables used for analysis. (XLSX)

S1 File Supplementary methods and tables. (PDF)

S2 File Supplementary figures. (DOCX)

S3 File Correlations between all variables used for analysis. (PDF)
