# Supplementary material for: Prosocial Behavior Increases with Age across Five Economic Games
Source: PLoS One. 2016 Jul 14;11(7):e0158671. doi: 10.1371/journal.pone.0158671 (PMC4945042; doi:10.1371/journal.pone.0158671)
Supplement: S1 File — (PDF) [file pone.0158671.s002.pdf]

Supplementary Methods and Tables

Sample

Six hundred non-student residents living in Machida, a suburban city of Tokyo, were selected from a list of about approximately 1,670 applicants who responded to a brochure distributed to approximately 180,000 households. These individuals (age range = 20–59 years, as of January 1, 2012) consisted of 75 men and 75 women in each 10-year age group. Of the 600, 564 actually participated in the initial wave of this study (May–July 2012) when demographic data were collected. One participant's responses to the demographic items showed obvious inconsistencies; therefore, this participant was excluded from the later waves of the study.

Among the 564 participants who participated in the initial wave, 483 returned for the second wave of the study (from October 2012 until February 2013) and participated in the first prisoner's dilemma game. The second prisoner's dilemma game and the first social dilemma game were conducted in the fourth wave (September–October 2013) with 474 participants. The dictator game was conducted in the third wave (April–June 2013) with 489 participants. The trust game was conducted in the fifth wave (from December 2013 until February 2014) with 471 participants. The second social dilemma game was conducted in the eighth wave (September–December 2015) with 424 participants. The triple dominance measure of the social value orientation (SVO) was administered in the third wave, the slider measure was administered in the fifth wave, and the ring measure was administered in the sixth wave (May–July 2014) with 470 participants.

Methods

Settings common to all game experiments

Four to ten people participated in one session, although they did not know how many others were actually in the laboratory, which consisted of 10 compartments. Participants were placed in individual compartments where they were visually, but not auditorily, isolated from the others. The instructions were displayed on each participant's computer screen and the participant read the instructions at his/her own pace. The instructions were written in Power Point with animated cartoons and accompanied by vocalization of the instructions to facilitate the understanding of the game. Participants wore headphones during the instruction phase.

Construction of the overall prosocial behavior measure

During study duration, 16 economic games were conducted (repeated one-shot prisoner's dilemma game with within-participant manipulation of the stake-size, one-shot prisoner's dilemma game with continuous choices, dictator game, faith game, trust game, risky dictator game, social dilemma game I, social dilemma game II, trucking game, stag-hunt game, ultimatum game, impunity game, second-party punishment game, third-party punishment game, social dilemma game with punishment, and pre-emptive strike game). From our measure of overall prosocial behavior, we first eliminated games where punishment was involved because the seemingly prosocial behavior in these games can be a purely strategic choice to avoid possible punishment. We also eliminated coordination and trust-related games because behaviors in those games was heavily influenced by the players' beliefs about other players' choices in addition to their prosociality. These criteria left us with 6 game behaviors (2 prisoner's dilemma games, 2 social dilemma games, a dictator game, and responder behavior in a trust game). The results of a factor analysis (number of participants who participated in all 6 games = 358; principal factor method) are shown in Table A, which indicates that these 6 game behaviors formed a single factor. The eigenvalue of the first factor (3.19) far exceeded the

eigenvalue of the second factor (0.28). Because the number of participants who played the second social dilemma game three and half years after the first wave was smaller (n = 424), reducing the number of participants who can be used in the analysis to 358, we decided to exclude this game in the following analysis. Another factor analysis excluding this game (n = 408) produced a similar result that is also shown in Table A. Furthermore, the correlation between the 5-game version and the 6-game version of the prosocial behavior measure was very high, r = 0.99. Therefore, we decided to use the remaining 5 game behaviors to construct the overall measure of prosocial behavior.

Table A. Factor structure of the game behaviors with and without the second social dilemma game

Game behavior	Loadings of the 1st factor	
	6 games	5 games
Prisoner's dilemma game, I	0.595	0.625
Prisoner's dilemma game, II	0.782	0.775
Dictator game	0.701	0.743
Social dilemma game, I	0.770	0.708
Social dilemma game, II	0.733	-
Return behavior in the trust game	0.773	0.810
Eigenvalue	3.186	2.700

Economic games used in the study

Prisoner's dilemma game, I: repeated one-shot game

The participants were endowed with either JPY 300, 800, or 1,500, which varied between trials, and they decided whether they were going to provide that endowment to their partner or keep it for themselves. When the endowment was provided, the partner received twice the amount of the endowment. For example, in a trial where the size of the endowment was JPY 1,500, the partner received JPY 3,000 if the participant gave the endowment to the partner. Similarly, the participant

received JPY 3,000 if the partner gave his/her endowment to the participant. When the participant did not provide and instead kept the endowment, he/she earned it.

Each participant played the game nine times. Of the nine games, each participant played the game with the simultaneous protocol three times, as the first player in the sequential protocol three times, and as the second player three times. In the simultaneous protocol, the two players made decisions without knowing the other party's choice. In the sequential protocol, one player (first player) makes a choice, and then the other player (second player) makes a choice after being informed of the first player's choice. The strategy method was used when the participant played as the second player; that is, the participant decided whether to provide or keep his/her endowment twice in each trial, assuming once that the first player had decided to provide, and once that the first player had decided not to provide. The outcome of the game was determined by the combination of the partner's actual choice and the participant's choice, given the partner's choice. Each participant played the game once for each combination of role and endowment size. The participants were instructed that three out of the nine games would be selected for actual payment. When all participants finished all nine games, one game was randomly selected from each stake size and used for actual payment. Each participant was randomly matched with another participant for each of the three games and paid for each game according to their actual choices. We used the proportion of trials that the participant provided his or her endowment to the randomly matched partner as an indicator of prosocial behavior in the first prisoner's dilemma game, excluding the participant's responses to the first player's defection in the second player trials because only very few of the participants cooperated in these trials.

Prisoner's dilemma game, II: one-shot game

Only the one-shot prisoner's dilemma game (PDG) with the simultaneous protocol was used. The participants were endowed with JPY 1,000 and decided how much of the endowment they were

going to provide to their partner in increments of JPY 100. When the endowment was provided, the partner received twice the amount of the endowment. For example, when the participant provided JPY 300, the partner received JPY 600. Similarly, the participant received JPY 1,200 if the partner provided JPY 600. The portion of the endowment the participant did not provide was his or hers to keep. We used the proportion of endowment the participant provided to his or her partner as an indicator of prosocial behavior in the second prisoner's dilemma game.

Dictator game

All the participants first played a one-shot dictator game as dictators who decided how to divide their endowment money, expecting that half of them would be assigned the role of recipients. Actually, each participant was paid as a dictator according to what he or she allocated to himself or herself and as a recipient according to what his or her randomly matched partner allocated to him or her. Each participant was given an endowment of JPY 1,000 and decided how much of the endowment to provide to their partner (the recipient). Following the initial dictator game, the participants played similar games six times as a dictator, with a different recipient each time. The size of the endowment varied each time, ranging from JPY 300 to JPY 1,300 (i.e., 300, 400, 600, 700, 1,200, and 1,300). Participants were told that they would play the game several times; however, they were not told how many times they would play the game. All participants made allocation decisions as dictator in each game knowing that two trials would be selected for actual payment. They were further informed that in one of the two games they would receive the money they allocated to themselves as dictator and that in the other game they would receive the money that the matched participant allocated to them. They were actually paid according to this scheme. In one of the two games, the participant played the role of a dictator, and in the other game the role of a recipient. We used twice the mean proportion of endowment that the participant allocated to his or her partners as an indicator of prosocial behavior in the dictator game. When the mean proportion exceeded .5, we set the participant's prosociality in

the dictator game at 1, based on the assumption that giving one-half of the endowment was considered prosocial. The additional analysis with the original score rather than the truncated score did not affect the conclusions.

Social dilemma game, I and II

The same design was used in the two social dilemma experiments. Participants were told that they would play the game in a group that the actual size was not conveyed. The instructions were written for a 10-person group, but the participants were told that the actual group size could vary. The game was played once and participants were paid based on their earnings in the game. Each participant was given an endowment of JPY 1,000 and decided how much of it to provide towards the production of a public good in increments of JPY 100. The sum of the provided money for the public good was doubled and equally allocated to all members regardless of their provision level. We used the proportion of the endowment that the participant provided as an indicator of prosocial behavior in the social dilemma game.

Trust game

The trust game was played between two randomly matched participants: a “truster” and a “trustee.” The truster was provided with JPY 1,000 by the experimenter and he/her decided how much of it to transfer to the trustee in increments of JPY 100. The transferred money was then tripled and provided to the trustee. The trustee then decided how much of the tripled money to transfer back to the truster. The endowment money of JPY 1,000 was provided only to the truster and not to the trustee, which differed from the standard version in which both players receive the same amount of endowment money. We introduced this feature to help the older non-student participants clearly understand that the back transfer of half of what they had received as a trustee resulted in a fair outcome when they were fully trusted by the truster. It is important to note that this is not the case in the standard trust or

investment game. When the trustee returns half of the transferred money after it has been transferred (e.g., when the truster transfers JPY 1,000 and the trustee returns JPY 1,500 (half of 3 times 1,000)), the truster receives JPY 1,500 and the trustee receives JPY 1,500 + endowment of JPY 1,000 = JPY 2,500. In the standard trust game, fairness depends on whether players focus only on the transferred money or include the original amount given to the trustee. All participants played as trusters and decided how much of the JPY 1,000 to transfer to the trustee, and then played as trustees and made decisions using the strategy method, without knowing which role they would be assigned. Then, pairs of participants were formed randomly, and one of each pair was randomly assigned either the truster's or the trustee's role, and received their payment according to the decisions of the pair. When they made decisions as trustees, the strategy method was used. That is, they were asked to indicate the amount of money they would transfer back to the truster in increments of 10% of the tripled money for each of the possible decisions of the matched truster (when they transferred JPY 100, 200, 300, 400, 500, 600, 700, 800, 900, and 1,000). Finally, pairs of participants were formed randomly, and one of each pair was randomly chosen as a truster and the other as a trustee. Each participant's earnings were determined according to their own and their partners' decisions in the assigned roles. We used the mean return proportion of the tripled money that participants transferred back as trustees as an indicator of prosocial behavior in the trust game. When the proportion exceeded .5, we set the participant's prosociality in the trust game as 1, based on the same logic we used in the analysis of the dictator game.

SVO measures

Triple dominance method

According to the triple dominance method (Van Lange, 1999), respondents were asked to imagine that they were paired with an anonymous partner, and then to choose one option from a set of three

options concerning how many points he/she and the other can earn. Respondents were also instructed to imagine that the other was also making the same set of decisions. The triple dominance measure consisted of 9 sets of choices (Table B) and those who made 6 or more consistent choices were categorized as prosocial, individualist, or competitor. If not, the SVO-type was recorded as missing. Then, individualists and competitors were both labeled as pro-selves.

Table B. Items of the triple dominance measure of SVO.

		YOU GET	OTHER GETS
Q1			
	OPTION1	480	80
	OPTION2	540	280
	OPTION3	480	480
Q2			
	OPTION1	560	300
	OPTION2	500	500
	OPTION3	500	100
Q3			
	OPTION1	520	520
	OPTION2	520	120
	OPTION3	580	320
Q4			
	OPTION1	500	100
	OPTION2	560	300
	OPTION3	490	490
Q5			
	OPTION1	560	300
	OPTION2	500	500
	OPTION3	490	90
Q6			
	OPTION1	500	500
	OPTION2	500	100
	OPTION3	570	300
Q7			
	OPTION1	510	510
	OPTION2	560	300
	OPTION3	510	110
Q8			
	OPTION1	550	300
	OPTION2	500	100
	OPTION3	500	500
Q9			
	OPTION1	480	100
	OPTION2	490	490
	OPTION3	540	300

In Q1–Q9, prosocial choices were options 3, 2, 1, 3, 2, 1, 1, 3, and 2; individualistic choices were options 2, 1, 3, 2, 1, 3, 2, 1, and 3; and competitive choices were options 1, 3, 2, 1, 3, 2, 3, 2, and 1.

Slider Method

According to the slider method, responders were asked to imagine that they were paired with another anonymous partner, and then they selected one of 9 options for allocating money (in JPY in the Japanese version) between the two persons. The slider measure consisted of 6 primary items (Q1–Q6) and 9 secondary items (Q7–Q15) listed below (Table C). The primary items were developed for assessing SVO prosociality while the secondary items were developed to disentangle the prosocial motivations of joint maximization from inequality aversion, which only applied for pro-socials.

Table C. Items of the slider measure of SVO.

		Option 1	Option 2	Option 3	Option 4	Option 5	Option 6	Option 7	Option 8	Option 9
Q1	You get	8500	8500	8500	8500	8500	8500	8500	8500	8500
	Other gets	8500	7600	6800	5900	5000	4100	3300	2400	1500
Q2	You get	8500	8700	8900	9100	9300	9400	9600	9800	10000
	Other gets	1500	1900	2400	2800	3300	3700	4100	4600	5000
Q3	You get	5000	5400	5900	6300	6800	7200	7600	8100	8500
	Other gets	10000	9800	9600	9400	9300	9100	8900	8700	8500
Q4	You get	5000	5400	5900	6300	6800	7200	7600	8100	8500
	Other gets	10000	8900	7900	6800	5800	4700	3600	2600	1500
Q5	You get	10000	9400	8800	8100	7500	6900	6300	5600	5000
	Other gets	5000	5600	6300	6900	7500	8100	8800	9400	10000
Q6	You get	10000	9800	9600	9400	9300	9100	8900	8700	8500
	Other gets	5000	5400	5900	6300	6800	7200	7600	8100	8500
Q7	You get	10000	9600	9300	8900	8500	8100	7800	7400	7000
	Other gets	5000	5600	6300	6900	7500	8100	8800	9400	10000
Q8	You get	9000	9100	9300	9400	9500	9600	9800	9900	10000
	Other gets	10000	9900	9800	9600	9500	9400	9300	9100	9000
Q9	You get	10000	9400	8800	8100	7500	6900	6300	5600	5000
	Other gets	7000	7400	7800	8100	8500	8900	9300	9600	10000
Q10	You get	10000	9900	9800	9600	9500	9400	9300	9100	9000
	Other gets	7000	7400	7800	8100	8500	8900	9300	9600	10000

Q11	You get	7000	7400	7800	8100	8500	8900	9300	9600	10000
	Other gets	10000	9600	9300	8900	8500	8100	7800	7400	7000
Q12	You get	5000	5600	6300	6900	7500	8100	8800	9400	10000
	Other gets	10000	9900	9800	9600	9500	9400	9300	9100	9000
Q13	You get	5000	5600	6300	6900	7500	8100	8800	9400	10000
	Other gets	10000	9400	8800	8100	7500	6900	6300	5600	5000
Q14	You get	10000	9600	9300	8900	8500	8100	7800	7400	7000
	Other gets	9000	9100	9300	9400	9500	9600	9800	9900	10000
Q15	You get	9000	9100	9300	9400	9500	9600	9800	9900	10000
	Other gets	10000	9400	8800	8100	7500	6900	6300	5600	5000

The slider measure of SVO was calculated from the answers of the 6 primary items following step 1–4 below (see Murphy, Ackermann, & Handgraaf, 2011 for more detail).

1. Calculate the mean of the payoffs a participant allocated to herself across the six primary items (As).

2. Calculate the mean of the payoffs a participant allocated to the other person across the six primary items (Ao).

3. Subtract 50 from both means: As−50 and Ao−50.

4. In order to compute the SVO angle, calculate the inverse tangent of the ratio of the mean of the payoffs allocated to the other minus 50 and the mean of the payoffs allocated to the self minus 50: $SVO^{\circ} = \arctan[(Ao - 50)/(As - 50)]$

In addition, individual participants' scores may be dichotomized to altruist, $SVO^{\circ} > 57.15^{\circ}$; prosocial, $22.45^{\circ} < SVO^{\circ} < 57.15^{\circ}$; individualist, $-12.04^{\circ} < SVO^{\circ} < 22.45^{\circ}$; competitor: $SVO^{\circ} < -12.04^{\circ}$. Altruist and prosocials are then re-categorized as prosocials, and individualist and competitors were re-categorized as proselfs.

The secondary items were used to measure unique features of prosociality (SVO angle between 22.45◦ and 57.15◦), using the 4 indices shown below. Note that these indices are valid only for prosocials.

1. Mean difference from archetypical inequality aversion). Options maximizing equality were 6, 5, 4, 7, 5, 8, 5, 3, and 2 in Q9–Q15, respectively.

2. Mean difference from archetypical joint gain maximization. Options maximizing joint gain were 9, [n/a], 1, 9, [n/a], 9, [n/a], 1, and 1 in Q9–Q15, respectively.

3. Mean difference from archetypical altruism. Options maximizing other's gain were 9, 1, 9, 9, 1, 1, 1, 9, and 1 in Q9–Q15, respectively.

4. Mean difference from archetypical individualism/competition. Options maximizing own gain were 1, 9, 1, 1, 9, 9, 9, 1, and 9 in Q9–Q15, respectively.

Ring Method

According to the ring method (Liebrand, 1984), participants were asked to imagine that they were paired with an anonymous partner, and then to choose one of a pair of options for the two to gain/lose. Participants were also instructed to imagine that the other was also making the same choices. The SVO ring measure consists of the following 24 items (Table D).

Table D. Items of the ring measure of SVO.

		You get	Other get
Q1	Option A	+ 390	-1450
	Option B	0	-1500
Q2	Option A	-1450	+ 390
	Option B	-1500	0
Q3	Option A	-1500	0
	Option B	-1450	-390
Q4	Option A	-1450	+ 390
	Option B	-1300	+ 750
Q5	Option A	-390	-1450
	Option B	-750	-1300
Q6	Option A	+ 750	+ 1300
	Option B	+ 1060	+ 1060
Q7	Option A	+ 750	-1300
	Option B	+ 1060	-1060
Q8	Option A	-1300	+ 750
	Option B	-1060	+ 1060
Q9	Option A	+ 1300	+ 750
	Option B	+ 1060	+ 1060
Q10	Option A	-1060	-1060
	Option B	-750	-1300
Q11	Option A	-1300	-750
	Option B	-1450	-390
Q12	Option A	-1060	+ 1060
	Option B	-750	+ 1300
Q13	Option A	+ 1450	- 390

	Option B	+ 1500	0
Q14	Option A	- 390	+ 1450
	Option B	- 750	+ 1300
Q15	Option A	+ 1300	- 750
	Option B	+ 1060	- 1060
Q16	Option A	- 390	+ 1450
	Option B	0	+ 1500
Q17	Option A	+ 1450	- 390
	Option B	+ 1300	- 750
Q18	Option A	+ 1450	+ 390
	Option B	+ 1300	+ 750
Q19	Option A	+ 390	- 1450
	Option B	+ 750	- 1300
Q20	Option A	+ 1450	+ 390
	Option B	+ 1500	0
Q21	Option A	+ 390	+ 1450
	Option B	+ 750	+ 1300
Q22	Option A	- 390	- 1450
	Option B	0	- 1500
Q23	Option A	- 1300	- 750
	Option B	- 1060	- 1060
Q24	Option A	+ 390	+ 1450
	Option B	0	+ 1500

The first step for computing the SVO ring measure is adding up the total gain for self and the other, and then obtaining the angle SVO◦ = arctan(other-gain/self-gain)*180/π (SVO◦ = 90 if the self-gain = 0). Participants were categorized into altruists (SVO◦ > = 67.5◦), pro-socials (22.5 < = SVO◦ < 67.5◦), individualists (-22.5◦ < = SVO◦ < 22.5◦), or competitors (SVO◦ < -22.5◦). To form a dichotomous categorization of pro-socials and proselfs, altruists and pro-socials were grouped together as pro-socials, and individualists and competitors were grouped together as pro-selves.

Satisfaction with the four outcomes of the PDG

In the post-experimental questions in each PDG experiment (PDG-I and PDG-II), participants were asked how happy they would feel if each of the following four events occurred in the PDG they played. Participants responded to each question on a 7-point scale (1 = *extremely unpleasant*, 2 = *unpleasant*, 3 = *somewhat unpleasant*, 4 = *neither unpleasant nor happy*, 5 = *somewhat happy*, 6 = *happy*, and 7 = *extremely happy*).

CC (mutual cooperation): "You and your partner provided money. As a consequence, both of you received twice as much money."

DC (unilateral defection by the participant): "You did not provide money and your partner provided money. As a consequence, you earned 3 times the endowment money and your partner earned nothing."

CD (unilateral cooperation by the participant): "You provided money and your partner did not provide money. As a consequence, you earned nothing and your partner earned three times the endowment money."

DD (mutual defection): "Neither you nor your partner provided money. As a consequence, both of you earned the original endowment money."

Beliefs in strategies for social success

Participants were asked to rate their response to a series of questions concerning strategies to succeed in life: "How important do you think the following items are as a means to be successful in life? Please choose one response for each item." A 7-point Likert-scale was used (1 = *not at all*, 2 = *not important*, 3 = *rather unimportant*, 4 = *neither unimportant nor important*, 5 = *rather important*, 6 = *important*, 7 = *very important*). The items were included as part of the questionnaires in waves 2 and 3 (Table E).

Table E. Items and factor loadings of the four subscales of the strategy of social success scale

Items	Loadings on the first factor
Manipulation subscale (α = 0.80)	
Taking advantage of others	0.698
Cheating others	0.599
Fooling people	0.823
Outwitting others	0.749
Showing off competence	0.490

Not being regarded lightly	0.453
Nepotism subscale (α = 0.82)	
Establishing strong relations with powerful people	0.560
Maintaining relations with various people who would help	0.685
Avoiding being disliked by other people	0.788
Accommodating the needs of other people by slightly	0.627
Being a person who is liked by other people	0.809
Assertiveness subscale (α = 0.83)	
Explicitly stating one’s own opinion	0.724
Taking leadership	0.722
Establishing one’s own point of view	0.777
Holding strong faith	0.682
Cultivating individuality	0.677
Carrying out plans in a rational manner	0.522
Honesty subscale (α = 0.75)	
Being honest	0.683
Not betraying others under any circumstances	0.785
Trusting others	0.754
Being a trustworthy person	0.474
Being considerate	0.383
Risk avoidance subscale (α = 0.66)	
Avoiding being exploited by others	0.701
Avoiding being fooled by others	0.929
Leading a stable life	0.423
Keeping away from dangerous places	0.372
Not aiming too high	0.381
