# Supplementary material for: Prosocial Behavior Increases with Age across Five Economic Games
Source: PLoS One. 2016 Jul 14;11(7):e0158671. doi: 10.1371/journal.pone.0158671 (PMC4945042; doi:10.1371/journal.pone.0158671)
Supplement: S2 File — (DOCX) [file pone.0158671.s003.docx]

**Supplementary Figures**

**Fig A.** **Gender and age**   **Fig B.** **Subjective social class**

**Fig C. Annual income in Japanese Yen Fig D. Number of participants with a 4-year college degree**

**Fig E. Marital status Fig F. House ownership**

**Fig G. Number of children Fig H. Number of siblings**

(including deceased)

**Figs A-H.** **Frequency distributions of gender, age (A), subjective social class (B), annual income in million Japanese Yen (JPY)**, N = 407 due to a missing response **(C), 4-year college degree (D), marital status (E), house ownership (F)**, N = 398 due to missing responses**, number of children (G), number of siblings** (including deceased) **(H).**

**Fig I**. **Frequency distribution of overall behavioral prosociality**. Each score on the horizontal axis indicates the level of the overall behavioral prosociality for a range of ±0.1. The overall behavioral prosociality is composed of the participants’ choices in five economic games, each of which was standardized first.
