# Supplementary material for: Prosocial Behavior Increases with Age across Five Economic Games
Source: PLoS One. 2016 Jul 14;11(7):e0158671. doi: 10.1371/journal.pone.0158671 (PMC4945042; doi:10.1371/journal.pone.0158671)
Supplement: S3 File — (PDF) [file pone.0158671.s004.pdf]

Correlations between all variables used in the analysis

	Beh prosoc	age	sex	SLM	TDM	RGM	CC	DC	CD	DD	Manipu-lation	Nepo-tism	Honest	Risk Avoid	Asser-tive	PD I	PD II	DG	SD I	SD II	TG	social class	incom e	college	Marital status	House Owner-ship	children
age	.28***<br>408																										
Sex (1=M)	-.04<br>408	.03<br>408																									
SLM	.66***<br>408	.17***<br>408	-.06<br>408																								
TDM	.43***<br>384	-.02<br>384	-.05<br>384	.47***<br>384																							
RNG	.39***<br>366	.04<br>366	.02<br>366	.42***<br>366	.33***<br>345																						
Satisfact'n with CC	.29***<br>408	-.06<br>408	-.02<br>408	.26***<br>408	.18***<br>384	.15**<br>366																					
Satisfact'n with DC	-.60***<br>408	-.34***<br>408	.20***<br>408	-.48***<br>408	-.33***<br>384	-.25***<br>366	-.19***<br>408																				
Satisfact'n with CD	.08<br>408	.05<br>408	.04<br>408	.10*<br>408	.09<br>384	.13*<br>366	-.22***<br>408	-.03<br>408																			
Satisfact'n with DD	-.31***<br>408	-.18***<br>408	-.11*<br>408	-.18***<br>408	-.11*<br>384	-.06<br>366	.02<br>408	.27***<br>408	.07<br>408																		
Manipula-tion	-.33***<br>408	-.24***<br>408	.07<br>408	-.28***<br>408	-.21***<br>384	-.15**<br>366	-.04<br>408	.30***<br>408	-.17***<br>408	.04<br>408																	
Nepotism	-.22***<br>408	-.21***<br>408	-.03<br>408	-.10*<br>408	-.08<br>384	-.17**<br>366	.07<br>408	.21***<br>408	-.10<br>408	.07<br>408	.30***<br>408																
Honesty	.17***<br>408	.11*<br>408	.06<br>408	.22***<br>408	.13*<br>384	.16**<br>366	.24***<br>408	-.16**<br>408	-.01<br>408	-.06<br>408	-.16***<br>408	.10*<br>408															
Risk avoidance	-.18***<br>408	-.03<br>408	-.19***<br>408	-.12*<br>408	-.19***<br>384	-.18***<br>366	-.05<br>408	.06<br>408	-.17***<br>408	.15**<br>408	.09<br>408	.30***<br>408	.050<br>408														
Assertive-ness	-.09<br>408	.01<br>408	.16**<br>408	-.06<br>408	-.04<br>384	-.08<br>366	.00<br>408	.06<br>408	-.06<br>408	.05<br>408	.12*<br>408	.24***<br>408	.27***<br>408	.08<br>408													
PD-I	.73***<br>408	.19***<br>408	-.01<br>408	.42***<br>408	.30***<br>384	.26***<br>366	.25***<br>408	-.53***<br>408	.08<br>408	-.25***<br>408	-.28***<br>408	-.19***<br>408	.13**<br>408	-.17***<br>408	-.05<br>408												
PD-II	.82***<br>408	.20***<br>408	.00<br>408	.46***<br>408	.31***<br>384	.29***<br>366	.25***<br>408	-.42***<br>408	.06<br>408	-.28***<br>408	-.21***<br>408	-.13**<br>408	.09<br>408	-.11*<br>408	-.07<br>408	.46***<br>408											
DG	.80***<br>408	.28***<br>408	-.13**<br>408	.60***<br>408	.41***<br>384	.32***<br>366	.17***<br>408	-.55***<br>408	.08<br>408	-.20***<br>408	-.32***<br>408	-.19***<br>408	.19***<br>408	-.13**<br>408	-.05<br>408	.51***<br>408	.51***<br>408										
SD-I	.78***<br>408	.15**<br>408	.05<br>408	.48***<br>408	.30***<br>384	.32***<br>366	.23***<br>408	-.35***<br>408	.09<br>408	-.27***<br>408	-.20***<br>408	-.17***<br>408	.10*<br>408	-.16**<br>408	-.09<br>408	.43***<br>408	.68***<br>408	.46***<br>408									
SD-II	.67***<br>352	.13*<br>352	.08<br>352	.48***<br>352	.33***<br>333	.29***<br>325	.28***<br>352	-.31***<br>352	.04<br>352	-.24***<br>352	-.18***<br>352	-.08<br>352	.16**<br>352	-.11*<br>352	-.08<br>352	.44***<br>352	.58***<br>352	.47***<br>352	.64***<br>352								
TG	.84***<br>408	.28***<br>408	-.08<br>408	.65***<br>408	.40***<br>384	.35***<br>366	.24***<br>408	-.50***<br>408	.00<br>408	-.21***<br>408	-.31***<br>408	-.18***<br>408	.18***<br>408	-.13**<br>408	-.09<br>408	.50***<br>408	.60***<br>408	.69***<br>408	.52***<br>408	.50***<br>352							
social class	.03<br>408	.22***<br>408	-.01<br>408	.02<br>408	.00<br>384	-.01<br>366	-.04<br>408	-.02<br>408	.06<br>408	-.07<br>408	-.10*<br>408	.03<br>408	.05<br>408	-.06<br>408	.06<br>408	-.06<br>408	.04<br>408	.04<br>408	.00<br>408	.03<br>352	.08<br>408						
Annual income	.03<br>407	.25***<br>407	.55***<br>407	-.05<br>407	-.07<br>383	.02<br>366	-.01<br>407	.10<br>407	-.04<br>407	-.12*<br>407	.05<br>407	-.02<br>407	.11*<br>407	-.16**<br>407	.21***<br>407	-.03<br>407	.05<br>407	.01<br>407	.06<br>407	.03<br>352	.03<br>407	.27***<br>407					
4-year college	.01<br>408	-.04<br>408	.12*<br>408	.02<br>408	.04<br>384	.00<br>366	.12*<br>408	.05<br>408	.04<br>408	-.10*<br>408	.05<br>408	.09<br>408	.08<br>408	-.10*<br>408	.08<br>408	-.03<br>408	.02<br>408	-.02<br>408	.03<br>408	.02<br>352	-.04<br>408	.16**<br>408	.18***<br>407				
Marital status	.14**<br>408	.49***<br>408	-.01<br>408	.09<br>408	.01<br>384	-.03<br>366	-.04<br>408	-.19***<br>408	-.03<br>408	-.15**<br>408	-.15**<br>408	-.05<br>408	.04<br>408	-.02<br>408	.020<br>408	.06<br>408	.14**<br>408	.11*<br>408	.06<br>408	.07<br>352	.20***<br>408	.36***<br>408	.17***<br>407	-.01<br>408			
House Ownership	.10<br>398	.45***<br>398	-.12*<br>398	.09<br>398	-.02<br>374	-.05<br>359	.00<br>398	-.19***<br>398	-.08<br>398	-.06<br>398	-.17***<br>398	.01<br>398	.09<br>398	.00<br>398	-.03<br>398	.06<br>398	.07<br>398	.12*<br>398	.02<br>398	.03<br>344	.13*<br>398	.33***<br>398	.09<br>398	.01<br>398	.53***<br>398		
Number of children	.12*<br>408	.52***<br>408	.01<br>408	.06<br>408	-.04<br>384	-.05<br>366	-.04<br>408	-.18***<br>408	-.03<br>408	-.08<br>408	-.11*<br>408	-.09<br>408	.00<br>408	-.05<br>408	.02<br>408	.07<br>408	.11*<br>408	.12*<br>408	.05<br>408	.05<br>352	.13**<br>408	.20***<br>408	.16**<br>407	-.12*<br>408	.54***<br>408	.40***<br>398	
Number of siblings	.05<br>408	.07<br>408	.04<br>408	-.01<br>408	.02<br>384	.10<br>366	-.04<br>408	-.07<br>408	.04<br>408	-.07<br>408	.01<br>408	-.04<br>408	.13*<br>408	-.03<br>408	.06<br>408	.11*<br>408	.02<br>408	.00<br>408	.00<br>408	.00<br>352	.06<br>408	.01<br>408	.08<br>407	-.16**<br>408	.03<br>408	.00<br>398	.04<br>408

*$P < .05$, **$P < .01$, ***$P < .001$; Lower entries are numbers of participants.
